# Assessing gluteus medius volume with freehand 3DUS: validating a practical imaging tool for complex muscle morphology

**DOI:** 10.1007/s11517-025-03503-x

**Published:** 2025-12-27

**Authors:** Ali Karimi Azandariani, Megan Gordon, Irene Kaiser, Oluwagbemiga DadeMatthews, Ali Mirjalili, Guillaume Spielmann, Hyun Kyung Kim

**Affiliations:** 1 School of Kinesiology, Louisiana State University, Baton Rouge, LA USA; 2 Anatomy and Medical Imaging, University of Auckland, Auckland, New Zealand

**Keywords:** 3D freehand ultrasound, Muscle volume, Reliability, Validity, Multiple sweeps

## Abstract

Accurate and accessible imaging techniques are essential for evaluating muscle morphology in both clinical and research settings. This study examined the validity and reliability of freehand three-dimensional ultrasound (3DUS) employing a multiple-sweep technique for measuring the volume of complexly shaped muscles such as the gluteus medius (GMed), with magnetic resonance imaging (MRI) as the reference standard. Twelve healthy participants (21.1 ± 1.5 years) underwent both 3DUS and MRI scans. Each GMed was scanned using three overlapping 3DUS sweeps, and two processors independently segmented all 3DUS and MRI images to calculate muscle volume. Agreement between 3DUS and MRI was evaluated using Bland-Altman plots, while intra- and inter-processor reliability for 3DUS were assessed using intraclass correlation coefficients (ICCs), coefficients of variation (CV%), typical error (TE), and minimal detectable change (MDC). The mean difference between 3DUS and MRI was minimal, with Bland-Altman plots demonstrating good agreement and no systematic bias. Inter- and intra-processor reliability were excellent (ICC = 0.972 and 0.999, respectively). A subgroup analysis (*n* = 10) comparing prone and side-lying positions using 3DUS also demonstrated good between-position reliability (ICC = 0.94). Freehand 3DUS with the multiple-sweep technique provides a valid, reliable, and practical alternative to MRI for measuring GMed muscle volume in both clinical and research applications.

**Graphical abstract:**

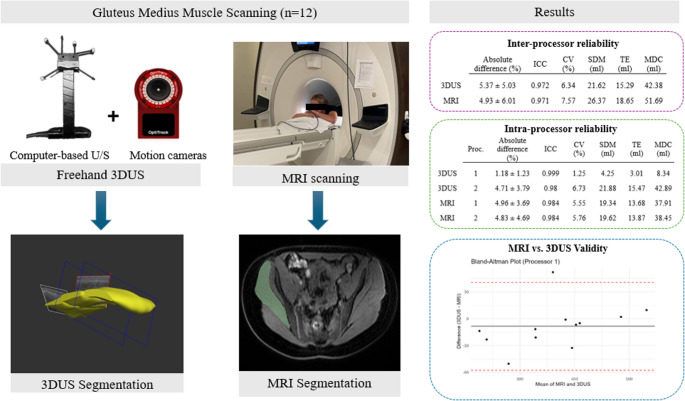

## Introduction

Muscle volume, a key indicator of muscle architecture, correlates linearly with muscle power and joint torque, and reflects both muscle growth and sarcomere number [1–3]. Several factors can influence muscle volume; for instance, aging typically reduces it [4], while treatments like surgery for pain alleviation and functional restoration can promote its recovery [5]. A previous study used muscle volume to predict muscle quality, defined as one repetition maximum divided by volume, in the context of aging and strength training [6]. Traumatic and congenital conditions can also cause asymmetries in muscle volume, potentially leading to functional imbalances and uneven joint loading; for example, individuals with congenital contralateral hip disease show reduced gluteus medius (GMed) volume on the affected side [7].

Freehand three-dimensional ultrasound (3DUS) provides a non-invasive method to assess muscle volume by synchronizing conventional B-mode ultrasound imaging with a motion tracking system to reconstruct a 3D model of the tissue [8]. This is typically achieved through a sweep approach, in which the operator moves the probe over the target muscle while positional data are recorded to reconstruct a volumetric image. While two-dimensional (2D) B-mode ultrasound is suitable for measurement of muscle cross-sectional area, fascicle length, and pennation angle, 3DUS enables measurement of muscle volume to evaluate morphological differences between healthy and pathological populations, or before and after different treatments [9, 10].

A validation study demonstrated that the single-sweep 3DUS technique can reliably assess the morphology of small muscles, such as the lateral gastrocnemius and flexor pollicis brevis, highlighting the importance of having a single acquirer to minimize variability across scans [11]. In addition to measuring muscle volume, 3DUS has also shown promise in capturing 3D surface shape, which helps determine the moment arm of a muscle based on its geometric path [12]. For larger muscles that exceed the transducer’s field of view, multiple-sweep techniques have been used successfully in both in vitro and in situ volume conditions [13]. Building on this approach, Barber et al. (2009) demonstrated strong agreement between 3DUS and magnetic resonance imaging (MRI) in measuring in vivo muscle volume of the medial gastrocnemius using the multiple-sweep technique [9]. This approach has also been successfully applied to muscles such as supraspinatus, infraspinatus, and posterior deltoid, showing close correspondence with MRI-based volume estimates [12].

Despite the growing use of 3DUS, most studies have focused on muscles with relatively linear fiber paths, such as the semitendinosus, vastus lateralis, and triceps surae [12]. In contrast, large, convergent muscles like GMed remain understudied. The GMed is clinically and functionally important but presents unique challenges for 3DUS due to its fan-shaped structure and width, ranging from 112.9 mm to 171.0 mm [3]. At least three sweeps are required to capture its full volume. Anatomically, the GMed serves as the primary abductor of the hip, and consists of anterior, middle, and posterior compartments, each with distinct functional roles [14, 15]. It helps stabilize the hip during gait and single-leg stance by limiting pelvic drop and maintaining frontal plane alignment [3]. Additionally, people with patellofemoral disease often show reduced GMed size compared to asymptomatic controls, potentially compromising hip abduction, extension, and lateral rotation [15]. However, despite its clinical and functional importance, the methodological feasibility of using 3DUS to measure GMed volume has not been well established.

Therefore, this study aimed to evaluate the validity and reliability of freehand 3DUS with a multiple-sweep technique, using MRI as the reference standard, for measuring GMed muscle volume. We hypothesized that freehand 3DUS using the multiple-sweep technique would demonstrate valid GMed volume measurements consistent with MRI and yield good reliability across processors.

## Methods

### Participants

Twelve healthy adults (4 females, 8 males; 21.1 ± 1.5 years; 173.5 ± 7.7 cm; 70.9 ± 13.9 kg; 23.37 ± 3.15 kg/m^2^) participated in this study. Participants were university students recruited through class announcements and flyers posted on the campus between September 2024 and April 2025. Inclusion criteria were adults aged 18–30 years with no neurological or lower-extremity diseases. Participants were excluded if they had any current or prior unrecovered lower extremity injuries, a high body mass index (BMI > 30), or any contraindications to MRI.

The minimum number of participants required for this study was calculated based on a previous study about 3DUS validation for muscle volume quantification [16]. A priori test power analysis was conducted based on the difference between two tests (1.9 cm³) and standard deviation (1.5 cm³). The minimum required sample size for this study was eight limbs to achieve 80% statistical power (0.8416) with a two-sided test (1.96). Additionally, the sample size was further supported by prior validation studies using 3DUS for muscle volume measurements, where sample size generally ranged from 10 to 18 [17, 18]. All procedures were performed in accordance with the 1964 Helsinki declaration and its later amendments. This study was approved by the Institutional Review Board, and all participants provided written informed consent before participating in the experiment.

### Experimental design

**Fig. 1:**
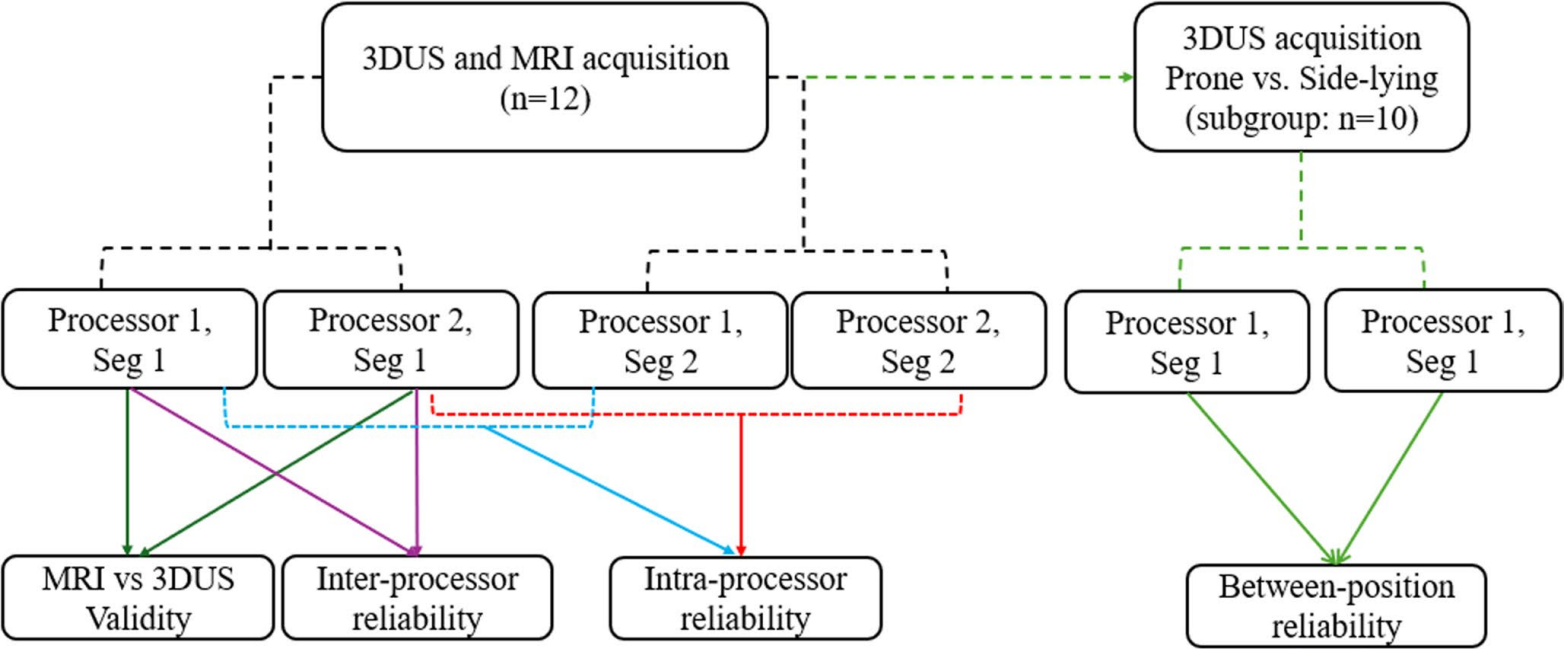
The study design for assessing the validity and reliability of freehand 3DUS measurements of GMed muscle volume. Each dataset was segmented independently by two processors to assess inter-processor reliability. For intra-processor reliability, processors repeated the segmentation 2-3 months after the initial segmentation. Validity was assessed by comparing 3DUS and MRI measurements. A supplementary analysis was conducted to examine the potential influence of body position (prone vs. side-lying) on GMed muscle volume measurements. Seg= segmentation

A repeated-measures, within-subject design was used in the current study (Fig. 1). All participants underwent both 3DUS and MRI scans on separate days, with an average of 11 days between sessions. Both limbs were scanned and processed, but only one limb per participant was randomly selected for further analysis to ensure data independence. To minimize the potential influence of exercise on muscle volume, participants were instructed to avoid intense hip-targeted resistance training during this period [19].

### Freehand 3DUS

**Fig. 2:**
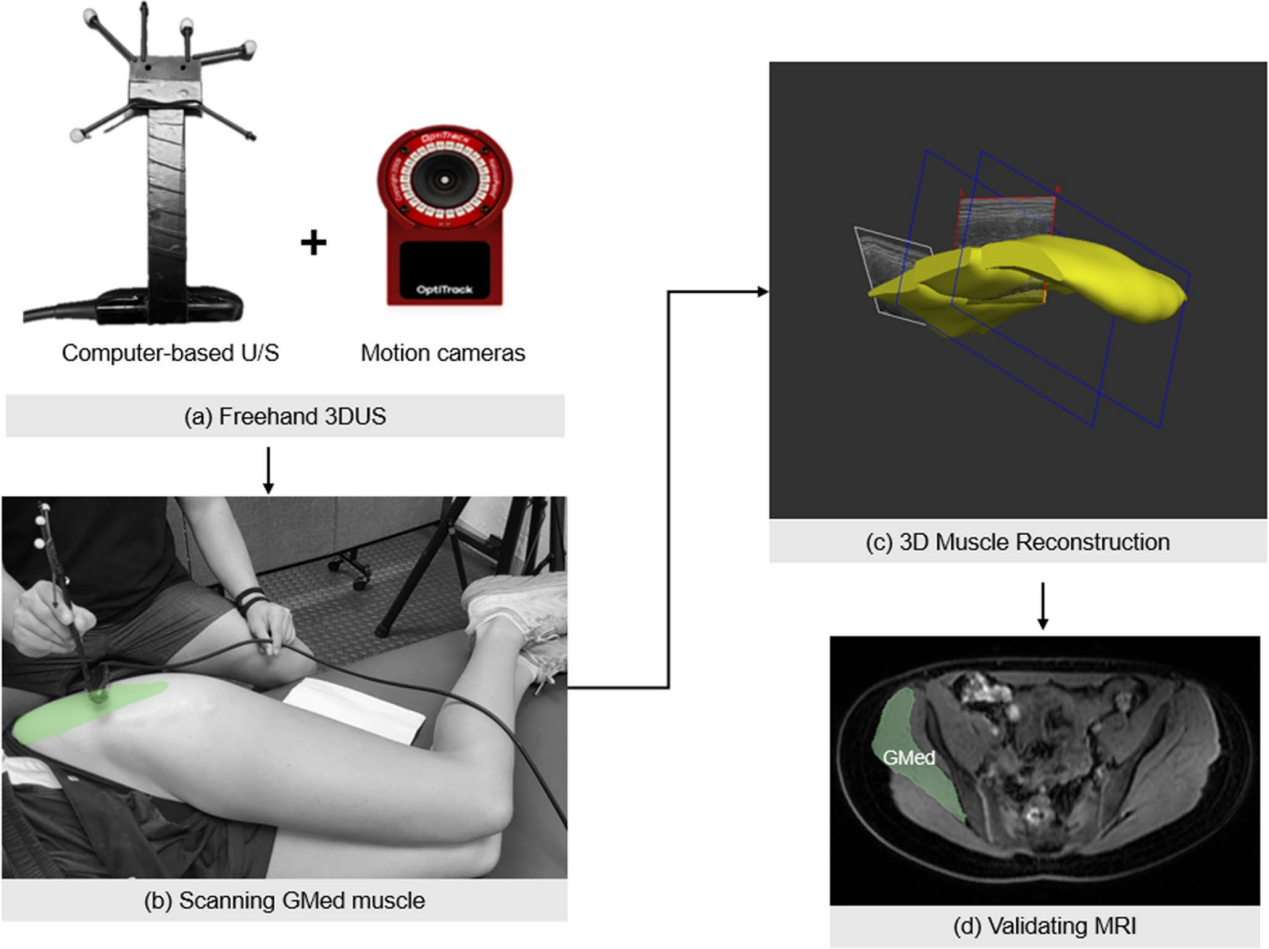
(**a**) Ultrasound device and motion capture cameras for tracking transducer position (**b**) The GMed muscle was scanned using a tracked ultrasound probe (**c**) 3D muscle reconstruction (**d**) A representative axial MRI slice with the GMed segmented in green

A computer-based B-mode ultrasound machine (MicrUs, TELEMED, Lithuania) (Fig. 2) with a 128-element beamformer linear transducer (field of view: 64 mm, depth: 60 mm, 8 MHz) was used to acquire ultrasound data via Stradwin software (v6.03, University of Cambridge, UK) [20]. To reconstruct a 3D model of the scanned muscle, the ultrasound machine was synchronized with four OptiTrack motion capture cameras (Flex 13, OptiTrack, Corvallis, OR, USA) to track the transducer’s position using four reflective markers placed on top of it. The 2D ultrasound system was calibrated by following the single-wall phantom calibration protocol in a water bath [21]. Before each session, the OptiTrack cameras were calibrated using a wand and a triangular ground plane calibration plate.

Participants were instructed to lie on their side with their hips naturally flexed to approximately 45 degrees, keeping their knees stacked one on top of the other (Fig. 2). They were also instructed to remain as still as possible during scans. Side-opening shorts were used to expose only the hip region, limiting unnecessary exposure of surrounding structures to ensure participants’ comfort. An acquirer (A.K.) with approximately one year of experience using freehand 3DUS scanned both legs five times each. Before scanning, the participant’s hip was palpated to identify the bony landmarks associated with GMed, including the anterior superior iliac spine (ASIS), iliac crest, and greater trochanter [22]. Multiple-sweep technique [13] was applied to each side of the hip, with each scan comprising three proximal-to-distal sweeps to capture the GMed. The first sweep began at the ASIS, the second sweep at the midpoint of the iliac crest, and the posterior sweep started at the posterior superior iliac spine (PSIS) [3, 22, 23]. Each sweep followed the muscle belly in a linear path and ended distally around the greater trochanter. Sufficient overlap was ensured between adjacent sweeps to allow accurate reconstruction of the full muscle volume.

Two independent processors, A.K (with approximately one year of experience) and I.K (with a minimum of 30 h of experience and no observation of the acquisition process), were trained by two medical doctors and anatomists (A.M and O.D) in muscle segmentation. They segmented the GMed by outlining the perimeter of the muscle in the axial plane every 20th to 30th frame of the B-mode 2-D ultrasound images. Each scan was labeled with a de-identified code prior to processing to minimize potential bias, and the segmentations were performed independently. Three trials with clear muscle boundaries and sufficient overlaps between sweeps were selected, and muscle volume (ml) was calculated by averaging the result of the three segmentations for each processor.

#### Prone vs. side-lying position

To assess whether participant posture influenced volumetric estimates, a supplementary analysis was conducted to examine the potential effect of body position on the GMed muscle volume measurements. A subset of ten participants (7 females, 3 males; 23.4 ± 4.3 years; 168.3 ± 6.8 cm; 64.85 ± 9.53 kg, 22.88 ± 3.16 kg/m^2^) underwent 3DUS scanning of the GMed in both prone and side-lying positions using the same multiple-sweep technique. The side-lying posture was identical to that described above. For the prone position, participants were instructed to relax their lower limb in a naturally extended position, with the ankle hanging freely off the edge of the assessment table to maintain a neutral angle. All scans were segmented by a single processor approximately two weeks after data acquisition. Each dataset was coded with anonymized labels so that the processor was blinded to both the participant’s position and identity to minimize potential bias.

### MRI

Participants were positioned prone on the MRI table to prevent soft tissue compression during weight-bearing and simulate the non-compressed situation of the muscle during 3DUS acquisition. Axial MRI scans were performed using a GE Discovery 750w 3.0T system (GE Healthcare, CA, USA) with a 16-channel surface array coil. The imaging protocol included T1 3D LAVA-FLEX sequence with the following parameters: spatial resolution = 1.7 × 2.5 × 3.4 mm, phase field of view = 0.6, frequency encoding = 300, phase encoding = 200, echo time (TE) = minimum, repetition time (TR) = 4 ms, with a flip angle of 12° and a total of 120 slices per slab.

Fat, water, and in/out-of-phase images were recorded from the iliac crest to the greater trochanter ensuring full coverage of the GMed boundaries [23]. To reconstruct the GMed 3D model and measure its volume, processors manually and independently segmented every 3rd to 5th MRI slice using the open-source 3D Slicer software (Version 5.9.0, Harvard University, Boston, MA, USA) [24] by drawing within the muscle’s perimeter (Fig. 2). For each processor, the muscle volume was calculated as the average of the three segmentations. The validity of 3DUS was assessed by comparing each processor’s 3DUS-based measurement to their corresponding MRI-based measurement.

### Statistical analysis

Both limbs were scanned and segmented; however, to avoid non-independence of bilateral observations, one limb per subject was randomly selected for statistical analysis. Statistical analysis of this study was performed using *R* (version 4.4.2, Posit Software, PBC, Boston, MA).Bland-Altman analysis, including calculation of themean difference and 95% limits of agreement [25] was utilizedto assess agreement between MRI and 3DUS measurements. Inter-processor reliability was tested by having two different processors (processor 1 and processor 2) segmenting the GMed on 3DUS and MRI data. Intra-processor reliability was evaluated by both processors segmenting the GMed on the same 3DUS and MRI data after 2–3 months. To assess inter- and intra-processor reliability of 3DUS and MRI segmentation, the Intraclass Correlation Coefficient (ICC; two-way mixed-effects model with absolute agreement) [26] was calculated with 95% confidence intervals (CI). Additionally, the coefficient of variation of the root-mean-square difference between repeated measurements (CV%) and typical error (TE) was calculated to evaluate variability relative to the mean [27]. TE (ml) was computed as $\:\frac{Standard\:deviation\:\left(SD\right)of\:the\:differences\:between\:measurements}{\sqrt{2}}$. The minimal detectable change (MDC) was also calculated as $\:1.96\:\times\:\:\sqrt{2\:\:}\:\times\:TE$ to indicate the smallest measurable change beyond measurement noise [26]. The standard deviation of the differences between measurements (SDM; ml) was calculated to describe the dispersion of individual values around the mean. To evaluate the influence of posture, GMed volumes from prone and side-lying position were compared. Agreement between postures was quantified using ICC (two-way mixed effects model with absolute agreement) and CV% calculated relative to the mean volume.

## Results

Freehand 3DUS and MRI scans of the GMed were acquired and processed for all participants, with one limb per participant randomly selected for analysis.

### Validity

**Fig. 3:**
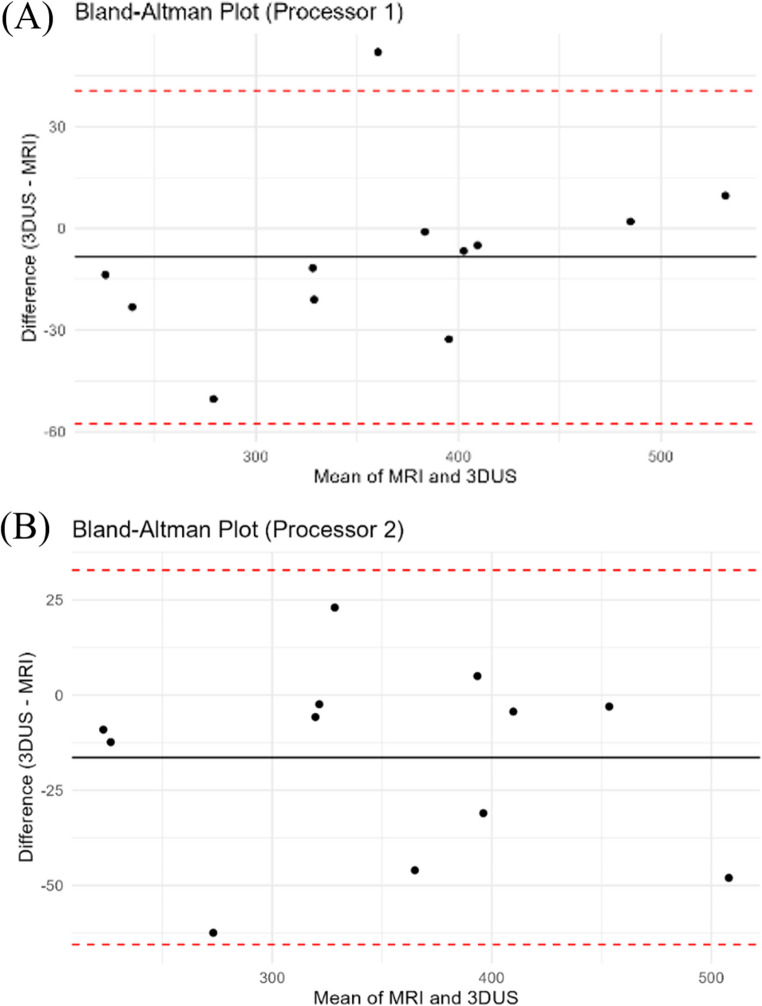
Bland-Altman plots comparing 3DUS and MRI muscle volume measurements for Processor 1 (**A**) and Processor 2 (**B**). The black line represents the mean difference, while the dashed red lines indicate the limits of agreement (±1.96 SD)

As the Bland-Altman plot shows, 3DUS and MRI demonstrated good agreement for GMed volume measurement, with most differences falling within the 95% limits of agreement (Fig. 3). For Processor 1, the mean difference between 3DUS and MRI was − 8.46 ± 25.07 ml, indicating a slight underestimation by 3DUS. The 95% limits of agreement ranged from 40.68 ml to −57.6 ml. For Processor 2, the mean difference was − 16.36 ± 25.08 ml, suggesting another slight underestimation by 3DUS. The 95% limits of agreement ranged from 32.8 ml to −65.52 ml.

### Inter-processor reliability

For 3DUS inter-processor reliability, the ICC was 0.972 (CI: 0.873–0.991), suggesting excellent reliability between measurements by Processors 1 and 2 (Table 1). The CV% was 6.34%, indicating minimal variability between processors. The mean difference between processors was 15.85 ml, meaning that Processor 1 produced slightly higher volume measurements than Processor 2. The standard deviation of differences between measurements (SDM) was 21.62 ml, indicating moderate variability relative to the average muscle volume. The TE was 15.29 ml, representing the expected average variability when comparing two processors’ measurements. The MDC was 42.38 ml, which means that a difference greater than this is likely to be a true difference rather than measurement noise. The absolute difference between processors was 5.37 ± 5.03%. Similarly, MRI segmentation also showed excellent inter-processor reliability (ICC = 0.971, CI: 0.922–0.989), with a CV% = 7.57%, mean difference = 7.87, SDM = 26.37 ml, TE = 18.65 ml, and MDC = 51.69 ml, absolute difference = 4.93 ± 6.01 (Table 1).

**Table 1:** GMed volume measurement for inter-processor reliability

	Seg1^a^ (ml)	Seg 2^b^ (ml)	Absolute difference (%)	ICC^c^	95% CI^d^	CV^e^ (%)	SDM^f^ (ml)	TE^g^ (ml)	MDC^h^ (ml)
3DUS	359.87 ± 97.35	343.62 ± 86.77	5.37 ± 5.03	0.972	0.84–0.97	6.34	21.62	15.29	42.38
MRI	368.33 ± 87.13	359.71 ± 89.03	4.93 ± 6.01	0.971	0.922–0.989	7.57	26.37	18.65	51.69

### Intra-processor reliability

For 3DUS, intra-processor reliability was higher than inter-processor reliability, with an ICC of 0.999 (CI: 0.997–0.999) and CV% of 1.25%, showing very high consistency for repeated measurements by Processor 1 after two months (Table 2). The mean difference between repeated segmentations was − 2.27 ml, showing minimal bias over time. SDM and TE were 4.25 ml and 3.01 ml, respectively. The MDC was 8.34 ml, meaning that changes greater than this number in repeated measurements by the same processor can be a real change rather than measurement noise. The absolute difference was 1.18 ± 1.23%, showing very low differences in repeated segmentations. Processor 2 also showed high intra-processor reliability of 3DUS with a mean difference of −1.65 ml, ICC of 0.98 (CI: 0.94–0.993), CV% of 6.73% and absolute difference of 4.71 ± 3.79% (Table 2). The SDM and TE were 21.88 ml and 15.47 ml respectively, and the MDC was 42.89 ml. Similar to 3DUS, MRI segmentation also demonstrated excellent intra-processor reliability for both processors with ICC of 0.984 (CI: 0.953–0.995) and CV% of approximately 5.7%. The mean difference between repeated segmentation was − 6.93 ml for Processor 1 and 3.62 ml for Processor 2 (Table 2). The SDM and TE were approximately 19–20 ml and 13–14 ml for both processors. The absolute difference of segmentations for Processor 1 was 4.96 ± 3.69% and that of Processor 2 was 4.83 ± 4.69%.

**Table 2:** GMed volume measurement for intra-processor reliability

	Processor	Seg1^a^ (ml)	Seg2^b^ (ml)	Absolute difference (%)	ICC^c^	95% CI^d^	CV^e^ (%)	SDM^f^ (ml)	TE^g^ (ml)	MDC^h^ (ml)
3DUS	1	359.9 ± 97.4	362.2 ± 98.6	1.18 ± 1.23	0.999	0.997–0.999	1.25	4.25	3.01	8.34
3DUS	2	343.61 ± 86.76	345.5 ± 85.32	4.71 ± 3.79	0.98	0.94–0.993	6.73	21.88	15.47	42.89
MRI	1	368.33 ± 87.13	375.09 ± 96.27	4.96 ± 3.69	0.984	0.953–0.995	5.55	19.34	13.68	37.91
MRI	2	359.71 ± 89.03	355.69 ± 90.63	4.83 ± 4.69	0.984	0.954–0.995	5.76	19.62	13.87	38.45

### Between-position reliability

The mean volume difference between prone and side-lying was − 6.2 mL (−2.0%). The ICC of 0.94 (CI: 0.79–0.99) and CV% of 5% indicated excellent between-position reliability, confirming that GMed volume measured by 3DUS was highly consistent between prone and side-lying position.

## Discussion

This study is the first to validate the use of 3DUS with a multiple-sweep technique for accurately measuring the volume of a complexly shaped muscle, the GMed, using MRI as the reference standard. The GMed’s broad origin and curved fiber orientation present a challenging target for accurate volume measurement, highlighting the need to validate advanced 3DUS multiple-sweep techniques. Our findings demonstrate that the 3DUS multiple-sweep approach yields valid and reliable volume estimates, with strong agreement compared to MRI and excellent intra- and inter-processor consistency. These results support the feasibility of using freehand 3DUS as a more accessible and cost-effective alternative to MRI for assessing muscle morphology in both clinical and research settings, particularly when repeated or large-scale measurements are needed.

Intra-processor reliability was excellent (ICC = 0.999 for Processor 1 and 0.98 for Processor 2), with a small MDC, suggesting that 3DUS is suitable for monitoring changes in GMed volume over time. A previous study [11] assessed intra-processor reliability using a 10-day gap between segmentations, while our study extended this to 60–90 days which reflects real-world intervals in clinical practice. Given the ICC of 0.972, inter-processor reliability in this study was also excellent, although variability was higher, likely due to the GMed’s complex structure and greater volume compared to smaller muscles like the lateral gastrocnemius (Bell et al. 2022). The MDC value indicates that changes greater than approximately 42 ml for both inter- and intra-processor assessments can represent physiological changes rather than measurement noise. Distinguishing between a physiological difference or measurement noise is particularly important in conditions such as patellofemoral osteoarthritis, where small changes may influence hip biomechanics [15].

**Fig. 4:**
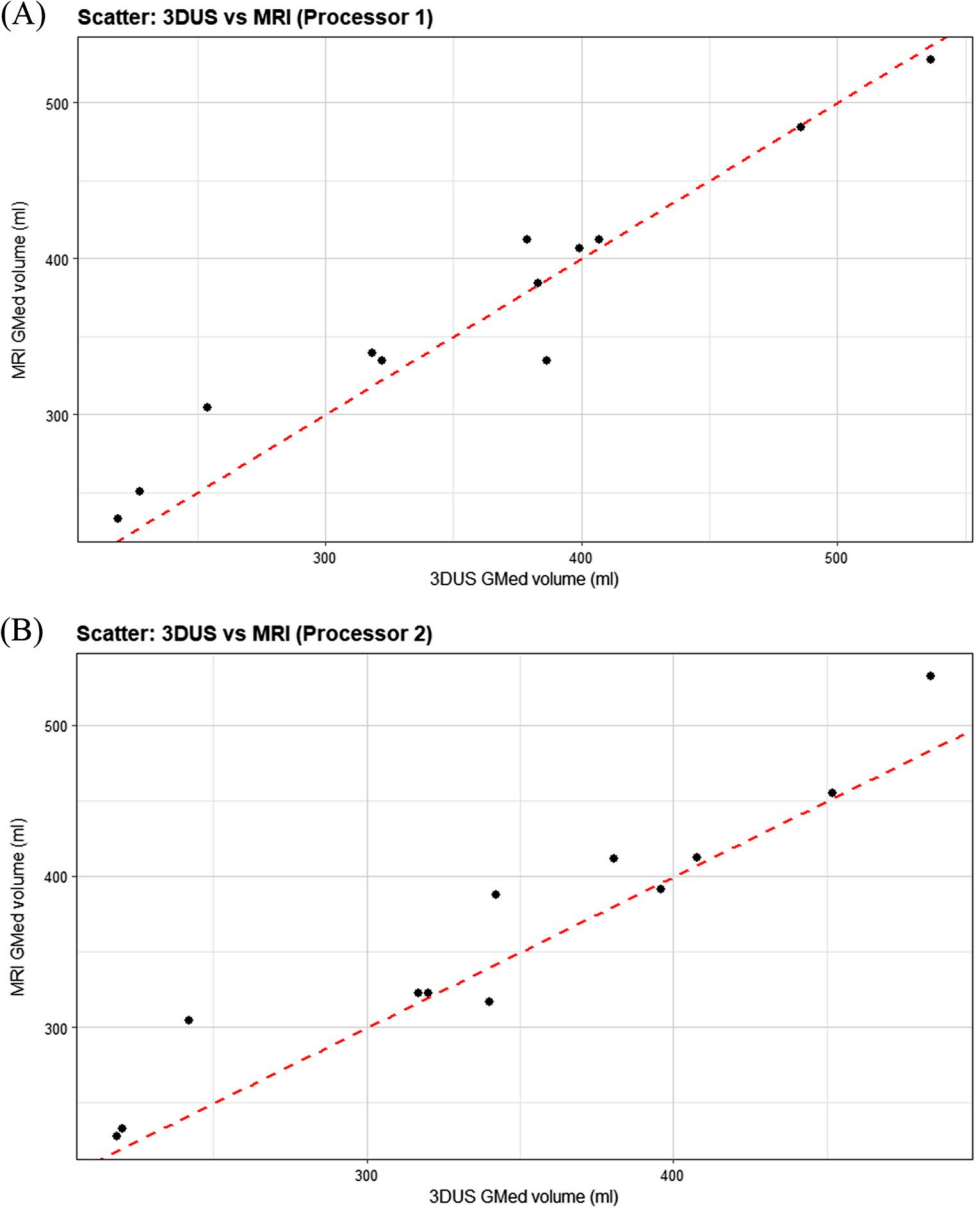
Scatter of GMed volume measured by 3DUS versus MRI for the analyzed limbs by Processor 1 (**A**) and Processor 2 (**B**). Each point represents one limb from one participant. The dashed red line indicates the line of identity (y = x)

**Fig. 5:**
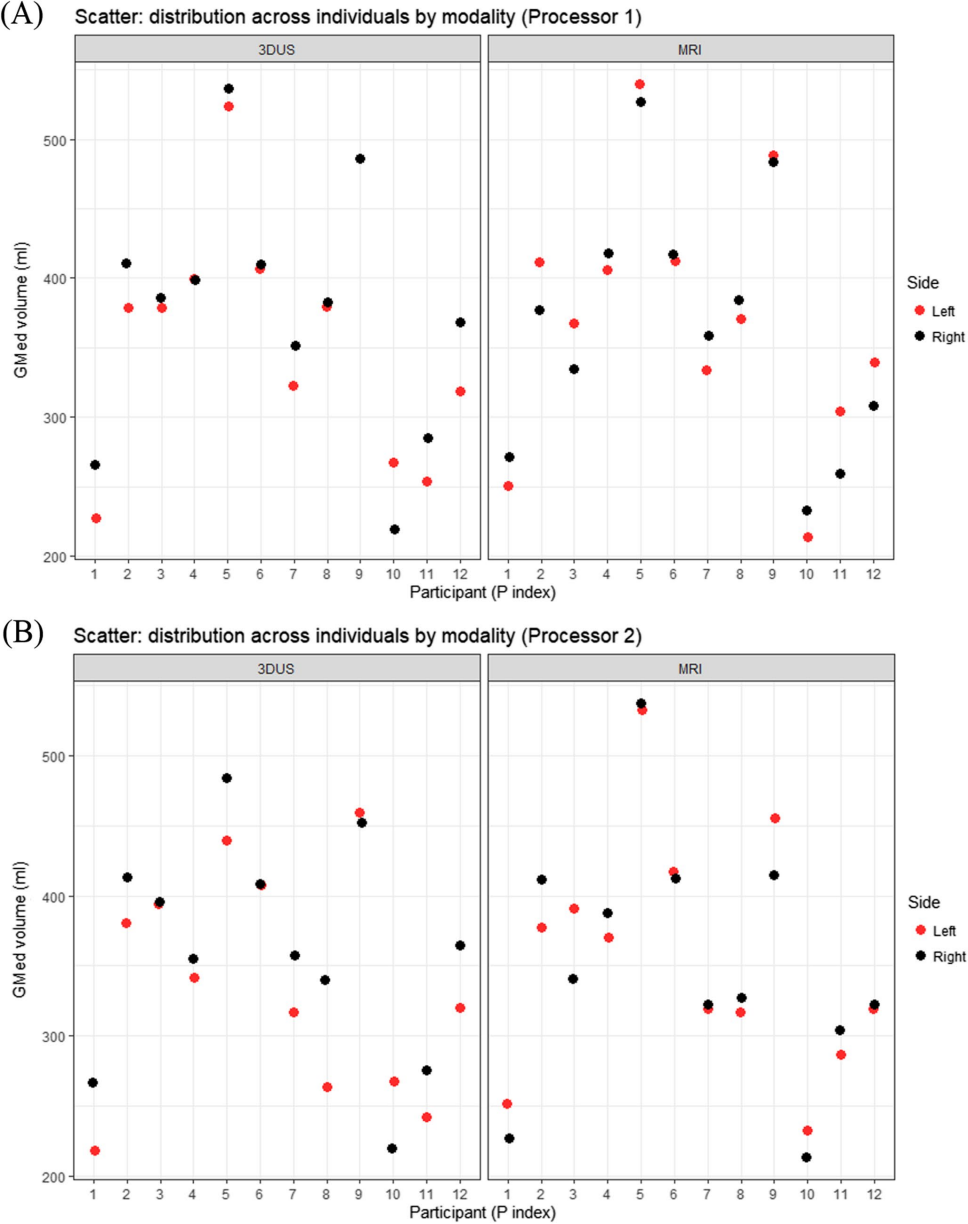
Scatter of GMed volume by participant by Processor 1 (**A**) and Processor 2 (**B**). Panels show 3DUS and MRI separately; each dot is one limb (red = left, black = right)

The mean difference observed between 3DUS and MRI measurements was small, indicating no significant bias. This finding is consistent with prior studies investigating the same validation on other lower extremity muscles [9, 11, 12]. Additionally, the scatter plots for each processor (Figs. 4 and 5) illustrate individual data distributions and further demonstrate the close relationship between 3DUS- and MRI-derived volumes, supporting the strong agreement observed in the Bland–Altman analysis. However, the limits of agreement for Processor 2 were wider than those for Processor 1, likely attributable to Processor 2’s less experience compared to Processor 1 and its lack of observation of the acquisition process [28]. Also, the Processor 1 intra-processor reliability was higher in 3DUS than MRI explained by their less experience in MRI segmentation than 3DUS (Table 2). This emphasizes the importance of experience, having a single acquirer [11] or potentially assigning the acquirer for the segmentation task as well.

Accurate segmentation is dependent on anatomy knowledge and clarity of the muscle boundaries [11]. One of the challenges for segmenting the GMed is distinguishing its boundary from the piriformis muscle [23] which was easier under 3DUS due to its superior magnification compared to MRI. While previous studies improved boundary definition using a water bath [9], this method is impractical for the gluteal region. Resolution enhancement is also possible by reducing the depth of penetration, but it is not possible due to thickness and depth of the GMed. An attempt made to potentially omit the transducer pressure on tissues was using a gel pad, which introduced handling challenges for the acquirer as controlling the slippery pad and the transducer simultaneously, was impossible during sweeps. Regarding coil pressure, most studies measuring gluteal or calf muscle volume have conducted MRI in the supine position [29–34]. In that position, the gluteal muscles inherently bear the weight of the pelvis and lower extremities, likely generating higher compressive forces than those applied by the surface coil. In contrast, our MRI acquisition was performed in the prone position to minimize tissue compression caused by body weight. The surface coil used in this study was light (55.8 × 66 cm, 3.5 kg) and rested on a 1–2 cm form spacer without straps of fixation, resulting in no visible tissue indentation (Fig. 2) [35]. Although it is not feasible to directly quantify coil pressure, the contact load was likely negligible compared to the gravitational load during supine positioning in previous MRI studies.

During ultrasound acquisition, the acquirer applied minimal transducer pressure (only sufficient for stable, gel-mediated contact) to avoid dropout artifacts, estimated at approximately 1 N [36]. To further examine the potential influence of posture, we conducted an additional test comparing GMed volume measured using 3DUS in the prone and side-lying positions. No significant difference was observed between positions (ICC = 0.94, CV = 5%), suggesting that moderate variations in posture had minimal influence on GMed volume under our controlled conditions. Similarly, Williams et al. (2022) [17] validated triceps surae volume assessment by 3DUS against MRI, despite different participant posture (prone for 3DUS, supine for MRI), supporting the robustness of muscle volume measurement across position. These results may suggest that maintaining a comparable limb configuration and minimizing probe compression are more important than matching an identical body posture for reliable muscle volume estimation. However, a related technical limitation in measuring GMed muscle volume is that the greater trochanter tends to appear more superficial and distinct in the side-lying position than in the prone position, which facilitates landmark identification and segmentation in 3DUS. Additionally, positioning the probe vertically during the first sweep (from the ASIS to the greater trochanter) in the prone position can be very challenging for the acquirer, as it is difficult to avoid friction with the bed or prevent the markers from being blocked by the cameras.

In addition, 3DUS offers practical advantages. Each muscle scan required approximately 5 min. Although MRI provides higher-resolution (but not necessarily clearer boundaries) and whole-body imaging, participants reported greater comfort and engagement during 3DUS, as the setup allowed them to view real-time visualization of their muscles on the screen. These logistical and experiential factors support the feasibility of 3DUS as a cost-effective alternative to MRI for muscle volume assessment [9, 18].

Despite all the benefits of freehand 3DUS, limited depth and field of view were critical limitations which prevented us from recruiting participants with excessive adipose tissue or particularly large gluteal muscles. To minimize potential confounding factors such as adiposity or age-related pathological conditions, we limited participants age to a younger range and included only individuals with a BMI below 30. This design allowed us to evaluate methodological performance of 3DUS without additional variability introduced by heterogeneity in tissue quality. Additionally, although the multiple-sweep technique helps address the limited field-of-view issue, only three sweeps were technically feasible due to software constraints. The reconstruction algorithm in Stradwin is designed to process only a small number of overlapping sweeps (typically up to three). In our pilot test, using more than three sweeps produced ghost boundaries that did not appear when limited to three sweeps. In Stradwin, the “dividers” are used to manage overlap regions. When we exceeded three sweeps, the dividers could no longer be positioned correctly between sweeps, making segmentation impossible in some cases. Based on these observations, we set an upper limit of three sweeps to prevent distortion in the final reconstructions. In practice, three overlapping, parallel sweeps cover the muscle mediolaterally; if a fourth sweep is needed, the person is usually large enough that the muscle is near the probe’s depth limit. Also, for more generalizability of the reliability result, inter- or intra-acquirer reliability needs to be assessed as well.

## Conclusion

This study confirms that 3DUS, using the multiple-sweep technique, shows good validity and reliability for GMed volume measurement. These findings demonstrate that 3DUS provides measurements that are consistent with MRI, segmentations by different processors, and repeated segmentation by the same processor over time. Thus, 3DUS can be accepted as an alternative for MRI for GMed volume measurement, especially when MRI is not accessible.

## Data Availability

The data supporting the findings of this study are available from the corresponding author upon reasonable request.
